# Scoring radiographic progression in ankylosing spondylitis: should we use the modified Stoke Ankylosing Spondylitis Spine Score (mSASSS) or the Radiographic Ankylosing Spondylitis Spinal Score (RASSS)?

**DOI:** 10.1186/ar4144

**Published:** 2013-01-17

**Authors:** Sofia Ramiro, Astrid van Tubergen, Carmen Stolwijk, Robert Landewé, Filip van de Bosch, Maxime Dougados, Désirée van der Heijde

**Affiliations:** 1 Department of Clinical Immunology and Rheumatology, Academic Medical Center, University of Amsterdam, Meibergdreef 9, 1100 DD Amsterdam, The Netherlands; 2 Department of Rheumatology, Hospital Garcia de Orta, Av. Prof. Torrado da Silva, 2801-951 Almada, Portugal; 3 Department of Medicine, Division of Rheumatology, Maastricht University Medical Center, P Debyelaan 25, 6229 HX Maastricht, The Netherlands; 4 School for Public Health and Primary Care (CAPHRI), University of Maastricht, P Debyelaan 25, 6229 HX Maastricht, The Netherlands; 5 Department of Rheumatology, Atrium Medical Center, Henri Dunantstraat 5, 6419 PC Heerlen, The Netherlands; 6 Department of Rheumatology, Ghent University Hospital, De Pintelaan 185, Ghent 9000, Belgium; 7 Department of Rheumatology B, Paris-Descartes University, Cochin Hospital, 27 rue du Faubourg Saint Jacques, 75014 Paris, France; 8 Department of Rheumatology, Leiden University Medical Center, Albinusdreef 2, 2333 ZA Leiden, The Netherlands

## Abstract

**Introduction:**

Radiographic damage is one of the core outcomes in axial SpA and is usually assessed with the modified Stoke Ankylosing Spondylitis (AS) Spine Score (mSASSS). Alternatively, the Radiographic AS Spinal Score (RASSS) is proposed, which includes the lower thoracic vertebrae, under the hypothesis that most progression occurs in these segments. We aimed to compare the mSASSS and RASSS with regard to performance.

**Methods:**

Two-yearly spinal radiographs from patients followed in the Outcome in AS International Study (OASIS) were used (scored independently by two readers). A total of 195 patients had at least one radiograph (12-year follow-up) to be included. We assessed the accessibility of vertebral corners (VCs) for scoring, as well as status and 2-year progression scores of both scoring methods. To assess the potential additional value of including the thoracic segment in the score, the relative contribution (in %) to the 2-year total RASSS progression of each spinal segment (cervical, thoracic and lumbar) was determined, and compared to the expected contribution, under the assumption that a balanced segmental progression would occur, proportional to the number of sites per segment.

**Results:**

The mSASSS could be scored in a total of 809 radiographs and the RASSS in 78% of these. In 58% of the latter, the score was based on one to two available thoracic VCs scores, and the remaining two to three were imputed because they were missing. There were 520 two-year mSASSS intervals available, and in 63% of them RASSS progression could be assessed. The mean (SD) 2-year interval progression score (330 intervals) was 2.0 (3.6) for the mSASSS and 2.4 (4.4) for the RASSS, yielding a similar effect size (mSASSS 0.57 and RASSS 0.55). Exclusive progression of the thoracic segment occurred in only 5% of the cases. There was no significant difference between the observed (14%) and expected (16%) contribution to progression of the thoracic segment (*P *= 0.70).

**Conclusions:**

The determination of RASSS for radiographic damage of the spine is frequently impossible or strongly influenced by non-contributory imputation. In comparison to the mSASSS, the contribution of thoracic VCs in the RASSS method is negligible, and does not justify the additional scoring efforts.

## Introduction

Radiographic damage is one of the core outcomes in axial spondyloarthritis (axial SpA) (including both non-radiographic axial SpA and ankylosing spondylitis (AS)) as recommended by the Assessment of SpondyloArthritis international Society (ASAS) [1]. Cross-sectionally, it is associated with impairment in spinal mobility [2,3] and longitudinally with functional disability [4], emphasizing the importance of assessment. ASAS recommends routine radiography of the lateral cervical and lumbar spine for assessing damage over time, but radiographs should not be repeated more frequently than every 2 years, unless indicated in individual cases, who might show faster progression [5,6].

Different scoring methods have been developed to quantify structural damage in axial spondyloarthritis: the Bath AS Radiology Index (BASRI) [7], the Stoke AS Spine Score (SASSS) [8] and a modification of the SASSS, the mSASSS [9]. In a formal comparison, the mSASSS has shown best reliability and sensitivity to change [10]. Consequently, it is the preferred scoring method for assessing structural damage in the spine for use in clinical trials, as endorsed by ASAS and Outcome Measures in Rheumatology Clinical Trials (OMERACT) [11]. The mSASSS assesses the presence of erosions, sclerosis, squaring, syndesmophytes and bridges at the anterior vertebral corners (VCs) of both the cervical and lumbar spine [9].

More recently, a new scoring method, the Radiographic AS Spinal Score (RASSS), has been proposed that includes the lower thoracic vertebrae, under the hypothesis that most progression is found in these segments [12]. Four thoracic VCs are added and the same features are scored as for the mSASSS, though with slightly modified scoring rules.

The usefulness of the RASSS has not been further evaluated thus far. Hence, it is important to compare both scoring methods, in order to establish the preferred method for the assessment of structural damage as an outcome measure.

Outcome measures should be valid in all their aspects. To standardize the nomenclature of validity, the OMERACT filter has been proposed and this includes three aspects: discrimination, truth and feasibility [13]. The main objective of the present study was to compare the mSASSS and RASSS with regard to performance, taking the aspects of the OMERACT filter into account.

## Materials and methods

### Patients and radiographs

Radiographs from patients included in the Outcome in Ankylosing Spondylitis International Study (OASIS) were used [14,15]. The OASIS study is a prevalence cohort including 217 consecutive patients with AS from the Netherlands, Belgium and France that started in 1996. According to protocol, cervical and lumbar spine radiographs were taken biannually for 12 years, with a total of seven possible time points per patient. For the present study, patients were included if they had at least one time point in which at least one of the radiographic damage scores could be calculated.

### Scoring methods

The two scoring methods used were the mSASSS [9] and the RASSS [12] (Table 1). In the mSASSS the anterior VCs of the cervical (lower border of C2 to upper border of T1) and lumbar (lower border of T12 to upper border of S1) segments (a total of 24 VCs) are scored at a lateral view, for the presence of erosion and/or sclerosis and/or squaring (1 point), syndesmophyte (2 points) and bridging syndesmophyte (3 points). The total score ranges from 0 to 72 [9]. The RASSS is similarly scored as the mSASSS with 3 modifications: 1) inclusion of the lower thoracic spine (lower border of T10 to upper border of T12; total of 28 VCs); 2) erosions are not scored; 3) squaring is not scored in the cervical spine. The RASSS ranges from 0 to 84 [12].

**Table 1:** Description of the mSASSS and RASSS scoring systems.

	mSASSS	RASSS
Spinal segments assessed		
- Cervical spine	Lower border of C2 to upper border of T1	Lower border of C2 to upper border of T1
- Thoracic spine	Not included	Lower border of T10 to upper border of T12
- Lumbar spine	Lower border of T12 to upper border of S1	Lower border of T12 to upper border of S1
Range of scoring system	0-72	0-84
Scoring definitions		
- 0	No change	No change
- 1	Erosion, squaring, sclerosis	Squaring only for the thoracic and lumbar segments; no erosions scored; sclerosis scores for all VCs
- 2	Syndesmophytes	Syndesmophytes
- 3	Bridging syndesmophytes/ankylosis	Bridging syndesmophytes/ankylosis

mSASSS, modified Stoke Ankylosing Spondylitis Spine Score; RASSS, radiographic Ankylosing Spondylitis Spinal Score; C, cervical; T, thoracic; S, sacral.

The radiographs were independently scored according to both scoring methods by two trained experts (SR and CS) who were blinded to demographic and clinical data. Both readers registered all the changes identified in each VC (for example, erosions, sclerosis and squaring) separately so that afterwards both scores could be computed. Because radiographs were taken in different formats during the 12 years of follow-up, enabling the readers to identify the points in time, they were scored with known chronology. All the available films per patient were scored at the same time.

Only scores of radiographs with ≤ 3 missing VCs per segment (either cervical or lumbar) were used. For the RASSS, the same rule applied and the four additional thoracic VCs were considered part of the lumbar segment [12]. Reliability between the two readers was explored using Bland and Altman analysis [16] on the progression intervals. All radiographs from patients with at least one score being beyond the 95% level of agreement were independently scored by an adjudicator (AvT). Averaged scores per VC of the two primary readers were used. In adjudicated cases, the score of the primary reader closest to the adjudicator was used. Missing VCs were imputed using an adaptation of the last-observation-carried-forward methodology. First, a missing value for a VC was replaced with the value of the previous observation. Then, the mean spinal segment's progression score (either cervical or lumbar) per patient was calculated. This was added to the imputed value, in an attempt to more accurately reproduce the true progression. This rule was applied assuring that the score achieved per VC never exceeded a score of 3. Similarly, in case of a score missing in a patient with a score of 0 in the same VC at a subsequent time point, the score of 0 for the previous time point(s) was assumed. If the baseline score of a VC was missing, the same procedure was applied, subtracting the mean segment progression from the score of year 2 for a particular patient. If a value of this VC was also missing at year 2, then an average of the other available VCs from this spinal segment at baseline was used to replace the missing VC(s).

Status and progression scores were calculated for both scoring methods. Status scores refer to the score in each of the available time points (at baseline and every 2 years thereafter). Progression scores were calculated as the difference between the status scores of two time points. Two-year progression scores refer to the progression occurring within 2 years, that is, status score of one time point minus the status score of the immediately previous time point. Twelve-year progression scores were computed as the score at year 12 minus the score at baseline.

### Use of the OMERACT filter to compare the scoring methods

The mSASSS and the RASSS were judged with respect to the different aspects of the OMERACT filter: truth, discrimination, and feasibility [13].

### Feasibility

The feasibility aspect of the OMERACT filter addresses the question: can the measure be applied easily, given constraints of time, money and interpretability? The feasibility of both methods (mSASSS and RASSS) was assessed. Because the RASSS requires a further four additional thoracic VCs to be present in the radiograph of the lumbar spine, the assessment of the ability to obtain both scores is important. The availability of the VCs and the ability to assess the status and the 2-year progression scores of both scoring methods was compared and the number of available VCs out of the four additional VCs included in the RASSS was also investigated. Comparisons were performed calculating a ratio of the available cases for the RASSS over the mSASSS, taking all radiographs into account, but also restricted to 1) patients with a RASSS available at year 12 in order to assess whether the RASSS would perform differently in the subset of patients with a complete follow-up and 2) patients with the first interval between years 0 and 2 available to compare with other results available in the literature for the RASSS [12].

### Discrimination

The discrimination aspect focuses on the question: does the measure discriminate between situations of interest? This aspect of the OMERACT filter pertains to sensitivity to change and reliability.

Inter-observer reliability was assessed for both status and progression scores for both mSASSS and RASSS, by means of Bland and Altman plots [16] and by calculation of the smallest detectable change (SDC) for each method. The SDC is the smallest change that can be detected beyond measurement error to determine change in an individual and was calculated as follows [17]: $\text{SDC}=\text{1}.\text{96}*\text{SD diff}/)\sqrt{k}*\sqrt{2}\;$. SD diff is the standard deviation (SD) of the set of differences in change scores obtained by two readers; k is the number of readers whose change scores are used (here: k = 2).

To obtain insight into sensitivity to change of the methods, the means and SDs of baseline, 2-year and 12-year status scores were assessed. Effect sizes (for all 2-year progression scores) were calculated for both mSASSS and RASSS dividing the mean value of the progression scores by the corresponding standard deviation.

### Truth

The truth aspect deals with the question: is the measure truthful, does it measure what is intended? Is the result unbiased and relevant? Both mSASSS and RASSS are, to a certain extent, similar, which means that they have a common part of construct validity. Therefore, we assessed the potential additional value of including the thoracic vertebrae in the RASSS, by determining the relative contribution (in %) to the 2-year total RASSS progression of each spinal segment (cervical, thoracic and lumbar) in comparison to the expected contribution. A balanced segmental progression, proportional to the number of VCs assessed in the RASSS (twelve cervical VCs, four thoracic VCs and twelve lumbar VCs) was assumed. The expected and balanced contribution assumed was 43% (12/28 VCs) for each of the cervical and lumbar segments and 14% (4/28 VCs) for the thoracic segment.

### Statistical analysis

Descriptive statistics were performed, with continuous variables being presented as mean (SD) and categorical variables as frequencies. Observed and expected progression rates were compared using the chi-square test and a 5% level of significance was assumed. Stata SE version 11 was used (Statacorp, College Station, TX, USA).

## Results

A total of 195 patients had at least one radiograph that could be scored (according to the mSASSS and/or RASSS), 64 had a radiograph that could be scored at year 12 and a total of 520 2-year progression intervals throughout the 12-year follow-up period were available Patients had a mean age of 42.8 (SD 12.4) years, mean disease duration since symptom onset of 20.0 (SD 11.6), mean disease duration since diagnosis of 11 (SD 8.7) years, 71% were males and 84% HLA-B27 positive. Baseline demographic, clinical and radiographic characteristics are summarized in Table 2.

**Table 2:** Baseline demographic, clinical and radiographic characteristics of the patients included in assessment of the radiographic progression in this study.

Assessment	*N *= 195*
Age (years)	42.8 (12.4)
Male gender (%)	138 (71%)
HLA-B27 positive (%)	158 (84%)
Symptoms duration (years)	20.4 (12.9)
Disease duration (years)	11.0 (8.7)
ASDAS-CRP	2.7 (1.0)
BASDAI (0-10)	3.4 (2.0)
BASFI (0-10)	3.2 (2.5)
BASMI (0-10)	3.7 (1.5)
CRP (mg/l) (*N *= 186)	17.5 (23.5)
Elevated CRP (%)‡	96 (52%)
mSASSS (0-72) (*N *= 177)¥	10.8 (15.2)
RASSS (0-84) (*N *= 130)	11.8 (16.6)
mSASSS of patients with available RASSS (0-72) (*N *= 130)	10.1 (14.2)
mSASSS > 0 (%) (*N *= 177)	143 (81%)
RASSS > 0 (%) (*N *= 130)	107 (82%)
mSASSS > 0 of patients with available RASSS (%) (*N *= 130)	106 (82%)

*Data are presented as mean (SD) or n (%). *N *= 195, representing patients with ≥ 1 radiograph with the mSASSS evaluable throughout follow-up (not necessarily at baseline available). ‡The cutoff was 10 mg/l for the Dutch patients (*n *= 122) and 5 mg/l for the Belgian (*n *= 23) and French patients (*n *= 50); ¥18 patients did not have a radiograph with an mSASSS evaluable at baseline, but had a radiograph in which the mSASSS could be calculated at a later time point and were therefore included in the study. ASDAS-CRP, Ankylosing Spondylitis Disease Activity Score (C-reactive protein); BASDAI, Bath Ankylosing Spondylitis Disease Activity Score; BASFI, Bath Ankylosing Spondylitis Functional Index; BASMI, Bath Ankylosing Spondylitis Metrology Index; CRP, C-reactive protein; mSASSS, modified Stoke Ankylosing Spondylitis Spine Score; RASSS, radiographic Ankylosing Spondylitis Spinal Score.

### Feasibility

The mSASSS could be scored in a total of 809 radiographs. The RASSS could be calculated in 78% of these radiographs (*n *= 629) (Tables 3 and 4). In 58% of those, in which the RASSS was calculated, the score was based on one or two available thoracic VC scores and the remaining two to three needed imputation because they were missing, so were in fact non-informative. There were 520 2-year mSASSS interval progression scores available, and in 63% of them a 2-year RASSS interval progression score could be determined. If the availability of all four additional VCs was required, then the RASSS could only be calculated in 226 (36%) radiographs and in 64 (19%) progression intervals. For the subgroups of radiographs from patients with the first 2-year interval available and of radiographs from patients with a RASSS available at year 12, see Tables 3 and 4.

**Table 3:** Feasibility of the mSASSS vs.

	All radiographs (*n *= 809)*			All radiographs from patients with RASSS available at year 12 (*n *= 341)*			All baseline radiographs from patients with baseline - year 2 interval available (*n *= 184)*		
	mSASSS	RASSS	% RASSS in radiographs with mSASSS	mSASSS	RASSS	%	mSASSS	RASSS	% RASSS in radiographs with mSASSS
Available status scores (≤ 3 missing VC per segment)	809	629	78%	341	277	81%	184	134	73%
Available 2-year progression intervals	520	330	63%	239	159	67%	164	96	59%

RASSS - availability of the status and progression scores.

*n = number of radiographs. mSASSS, modified Stoke Ankylosing Spondylitis Spine Score; RASSS, radiographic Ankylosing Spondylitis Spinal Score; VC, vertebral corner.

**Table 4:** Feasibility of the RASSS - availability of the four thoracic vertebral corners added to the RASSS.

Availability of the four VCs added in the RASSS	All radiographs with RASSS evaluable (*n *= 629)*		All radiographs from patients with RASSS available at year 12 (*n *= 341)*		All baseline radiographs from patients with baseline - year 2 RASSS interval available (*n *= 134)*	
	N	%	N	%	n	%
- 1 VC only	65	10%	28	10%	18	13%
- 2 VCs	297	47%	139	50%	71	53%
- 3 VCs	41	7%	18	7%	11	8%
- 4 VCs	226	36%	92	33%	34	25%

*n = number of radiographs. mSASSS, modified Stoke Ankylosing Spondylitis Spine Score; RASSS, radiographic Ankylosing Spondylitis Spinal Score; VC, vertebral corner.

### Discrimination

**Figure 1:**
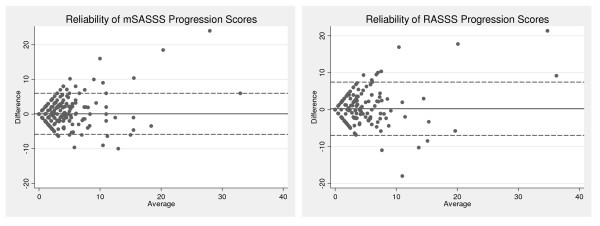
**Bland and Altman plots: reliability of the mSASSS and RASSS progression scores**. Difference against mean for mSASSS and RASSS progression scores of the two readers. The SDC for the progression scores was 2.9 for the mSASSS and 3.5 for the RASSS. mSASSS, modified Stoke Ankylosing Spondylitis Spine Score; RASSS, Radiographic Ankylosing Spondylitis Spinal Score; SDC, smallest detectable change.

The first part of the discrimination aspect is reliability. Figure 1 shows the Bland and Altman plots for the progression scores of both the mSASSS and RASSS. In general, both scores could be reliably performed without clear systematic error. The SDC for the progression scores was 2.9 for the mSASSS and 3.5 for the RASSS.

Of all radiographs in which both the mSASSS and RASSS could be determined (*n *= 629), the mean (SD) status score was 15.5 (17.9) units for the mSASSS and 18.0 (20.9) units for the RASSS (Table 5). The mean (SD) 2-year progression score, calculated in 330 2-year intervals, was 2.0 (3.6) for the mSASSS and 2.4 (4.4) for the RASSS. The progression score per spinal segment was 1.2 (2.3) for the cervical segment, 0.8 (2.1) for the lumbar segment and 0.4 (1.2) for the thoracic segment in the RASSS. The effect size of the 2-year progression score was 0.57 (2.0/3.6) for the mSASSS and 0.55 (2.4/4.4) for the RASSS.

**Table 5:** Status and progression mSASSS and RASSS scores for radiographs in which RASSS was evaluable.

	All radiographs with RASSS evaluable (*n *= 629)		All radiographs with RASSS evaluable and from patients with RASSS available at year 12 (*n *= 277)		All radiographs with four additional thoracic VCs available (*n *= 226)		All baseline radiographs with RASSS evaluable from patients with baseline-year 2 interval evaluable (*n *= 134)	
**STATUS SCORES**	**mSASSS**	**RASSS**	**mSASSS**	**RASSS**	**mSASSS**	**RASSS**	**mSASSS**	**RASSS**
Total Score	15.5 (17.9)	18.0 (20.9)	18.0 (19.3)	20.8 (22.4)	17.3 (18.2)	20.5 (21.4)	10.2 (14.2)	11.8 (16.6)
Cervical segment	8.4 (10.1)	7.9 (10.1)	10.0 (10.7)	9.5 (10.7)	8.9 (10.0)	8.3 (10.0)	5.5 (8.2)	5.2 (8.1)
Lumbar segment	7.1 (9.8)	7.1 (9.8)	8.0 (10.0)	8.0 (9.9)	8.4 (10.2)	8.4 (10.2)	4.6 (7.9)	4.6 (7.9)
Lumbar segment (with thoracic segment included)	-	10.1 (13.1)	-	11.4 (13.2)	-	12.2 (13.7)	-	6.6 (10.7)
Thoracic segment	-	3.0 (3.9)	-	3.4 (3.8)	-	3.8 (4.4)	-	2.0 (3.4)

2-YEAR PROGRESSION SCORES	All radiographs with 2-year RASSS intervals evaluable (*n *= 330)		All radiographs with RASSS evaluable and from patients with RASSS available at year 12 and with 2-year RASSS intervals evaluable (*n *= 159)		All radiographs with four additional VCs available and 2-year RASSS intervals evaluable (*n *= 64)		All radiographs with RASSS evaluable from patients with baseline-year 2 interval evaluable (*n *= 96)	
Total Score	2.0 (3.6)	2.4 (4.4)	2.4 (4.5)	2.9 (5.5)	2.1 (3.3)	2.8 (4.2)	1.9 (4.1)	2.2 (5.0)
Cervical segment	1.2 (2.2)	1.2 (2.3)	1.5 (2.6)	1.4 (2.9)	1.2 (2.0)	1.1 (1.9)	1.0 (2.6)	0.9 (2.9)
Lumbar segment	0.8 (2.0)	0.8 (2.1)	1.0 (2.4)	1.0 (2.4)	0.9 (2.2)	0.9 (2.2)	0.9 (2.2)	0.9 (2.2)
Lumbar segment (with thoracic segment included)	-	1.2 (2.8)	-	1.5 (3.3)	-	1.6 (3.3)	-	1.3 (3.1)
Thoracic segment	-	0.4 (1.2)	-	0.5 (1.2)	-	0.7 (1.7)	-	0.4 (1.4)

*Data are expressed as mean (SD). mSASSS, modified Stoke Ankylosing Spondylitis Spine Score; RASSS, radiographic Ankylosing Spondylitis Spinal Score; SD, standard deviation, VC, vertebral corner.

For all radiographs from patients with a RASSS evaluable at 12-year follow-up (*n *= 59), the mean (SD) status score of the mSASSS was 18.0 (19.3) and of the RASSS 20.8 (22.4); and in this group the mean (SD) 2-year progression score of the mSASSS was 2.4 (4.5) and of RASSS 2.9 (5.5). Compared to the status scores of all radiographs, the status scores of the radiographs from both patients with a RASSS evaluable at 12-year follow-up and radiographs with the four additional thoracic VCs available were higher (Table 5). During the first 2-year follow-up, the progression was 1.9 (4.1) units for the mSASSS and 2.2 (5.0) units for the RASSS in patients with available intervals for both mSASSS and RASSS (*n *= 134).

In patients with an mSASSS evaluable at baseline and at 12-year follow-up (*n *= 64), the mean (SD) 12-year progression was 11.7 (11.5). In 38 out of the 64 patients, the RASSS could be assessed with a mean 12-year progression of 14.2 (14.6) and a corresponding mSASSS progression of 12.2 (12.5) in this group.

In patients with a RASSS available at year 12 (*n *= 59), the baseline, 2-year and 12-year RASSS status scores were 11.8 (15.3), 12.3 (13.9) and 28.5 (25.0), respectively. For the mSASSS, the corresponding status scores were 10.6 (13.6), 11.2 (12.4) and 24.5 (21.6), respectively.

Interestingly, in a few cases, the RASSS enabled the occurrence of negative progression scores. Taking all radiographs into account, in five VCs in the cervical segment, first sclerosis was considered to be present and scored as 1, but at follow-up an erosion appeared, which was still scored as a 1 in the mSASSS but was scored 0 according to the RASSS scoring rules and, consequently, the progression score for that specific VC was -1 for RASSS.

### Truth

Exclusive progression in the thoracic segment, which can only be captured by the RASSS and not by the mSASSS, occurred in only 5% of the 2-year progression intervals (17 intervals out of 330). In 2% of the 2-year progression intervals, a progression of two or more units (possibly indicating new syndesmophyte formation) in the thoracic segment was found. In 25% of the intervals (81 out of 330 intervals), progression occurred exclusively in the cervical segment and in 7% (23 out of 330) in the lumbar segment. However, there were no significant differences between the observed and expected contributions of the thoracic segment to progression (16% vs. 14%, *P *= 0.70), whilst progression was observed more frequently than expected in the cervical spine (55% vs. 43%, *P *= 0.09), and less frequently in the lumbar spine (29% vs. 43%, *P *= 0.04) (Table 6).

**Table 6:** Ratio of 2-year progression in each of the spinal segments of the RASSS.

	Relative contribution in each of the spinal segments of the RASSS (in %)		*P *value for the difference
		
	Expected	Observed	
Cervical segment (12 VCs)	43%	55%	0.09

Lumbar segment (12 VCs)	43%	29%	0.04

Thoracic segment (4 VCs)	14%	16%	0.70

*n = 330 represents progression intervals. RASSS, radiographic Ankylosing Spondylitis Spinal Score; NA, not applicable.

## Discussion

The present study shows that the mSASSS remains the most appropriate method for scoring radiographic progression in patients with AS based on feasibility, discrimination and truth aspects of the OMERACT filter.

With regard to feasibility, the 2-year progression scores were available for the RASSS in only 63% of the cases in which mSASSS progression intervals could be calculated. In the paper describing the RASSS, the authors reported an availability of 88% of the progression scores within the first 2 years [12], while in our study only in 59% of the cases a RASSS progression score could be calculated in this first 2-year interval. Furthermore, in our study in one third of the radiographs in which the RASSS could be calculated, only one to two thoracic VCs were accessible, meaning that calculation of the RASSS was based on imputed and therefore non-informative VCs in the lumbar spine. This shows that an important number of radiographs obtained in the Netherlands, France and Belgium does not include the lower part of the thoracic spine, in contrast to what has been suggested for Germany [12]. According to the OMERACT filter, feasibility captures an essential element in the selection of measures, one that may be decisive in determining a measure's success [13]. The worse feasibility of the RASSS compared with the mSASSS jeopardizes its wide use.

The RASSS demonstrated a higher mean progression, but an increase in the variance of the progression scores was also observed, resulting in similar effect sizes between mSASSS and RASSS. A higher mean progression was expected because the RASSS includes four additional VCs compared with the mSASSS, so that the RASSS is by definition almost always higher than the mSASSS. Exceptions are only the cases with erosions in any segment or squaring in the cervical spine, which are scored for the mSASSS, but not for the RASSS. Nevertheless, and comparing the progression scores with the limited data available in the literature for the RASSS, our RASSS progression scores were higher. In the first 2 years, we found a progression in the RASSS of 2.2 (5.0), whereas Baraliakos *et al*. reported a 2-year progression of 1.6 (2.8) [12]. Also, our mSASSS progression scores were higher compared with the same study (1.9 (4.1) vs. 0.9 (2.5)) [12]. A possible explanation for the difference in progression scores can be the difference in baseline radiographic damage (baseline mSASSS of 8.1 (14.6) in German cohort vs. 10.2 (14.2) in OASIS cohort). It is well known that presence of radiographic damage is a predictor of further and faster progression of radiographic damage [18-20]. Other literature on RASSS progression scores is currently lacking. However, our mSASSS progression scores can be compared with other available studies. There are reports of 2-year mSASSS progression scores of around 1 mSASSS unit [21-23], 2.5 units [18] and 2.6 units (extrapolation to a 2-year period of the annual progression rate of 1.3 (2.5), and assuming linearity) [20]. The differences between scores can be attributed to differences in selection of patients, baseline radiographic damage of patients, conditions in which radiographs were read [24] or the method of imputation of missing VCs. The increase in the variance around the progression scores resulted in similar effect sizes for both methods (0.57 for the mSASSS and 0.55 for the RASSS), showing that the higher mean progression of the RASSS is offset by the increased noise. For discrimination, both scoring methods seemed to be reliable, however, the SDC for the mSASSS was slightly smaller compared to the RASSS (2.9 vs. 3.5), suggesting that the measurement error with the RASSS is somewhat higher. Reliability of the RASSS could possibly improve by having an additional and separate radiograph to score the thoracic VCs. This could reduce the parallax associated with extending the view of the lumbar radiograph to include the thoracic VCs, but would on the other hand imply higher costs and radiation for the patients. With regard to the truth aspect of the OMERACT filter, we found that most progression occurred in the cervical segment of the spine (55%), followed by the lumbar spine (29%) and only 16% was found in the thoracic vertebrae. Furthermore, the progression in the thoracic vertebrae was not significantly different from what was expected, if progression throughout the spine would occur in a balanced way. In addition, we showed that new syndesmophytes exclusively occurring in the thoracic spine occurred in a maximum of 2% of the intervals. These data should be interpreted with caution, because a progression score of 2 does not always correspond to a new syndesmophyte, but can also mean twice a score of 1 in two separate VCs, reflecting development of squaring or sclerosis. This shows that the RASSS does not capture more progression occurring in the thoracic vertebrae, as was hypothesized by Baraliakos *et al. *[12]. In our study, a 2-year progression in the thoracic vertebrae of 0.4 (1.4) out of a total RASSS progression of 2.2 (5.0)) was found. Baraliakos *et al*. reported a progression of 0.6 (3.3) out of a total RASSS progression of 1.6 (2.8), indicating a higher contribution from the thoracic VCs to the total RASSS. The reason for this discrepancy between both studies is not entirely clear, but availability of the thoracic VCs for scoring can play a role.

Some limitations of the present study should be addressed. Films were obtained throughout a 12-year follow-up, which means that some of them were old and did not have the optimal quality. Nevertheless, this limited the reading of both scoring methods similarly. Furthermore, the findings of this study may not be generalizable to countries where lumbar radiographs routinely include the low thoracic spine, which is not standard procedure in our three countries.

## Conclusions

In conclusion, the calculation of RASSS for status or progression of radiographic abnormalities in the spine is frequently impossible or strongly influenced by non-contributory imputation. The effect size of both methods is similar. In comparison to the mSASSS, the contribution of thoracic VCs in the RASSS is negligible, and does not justify the additional scoring efforts. The mSASSS remains the most appropriate measure to assess radiographic damage in patients with AS.

## Abbreviations

AS: Ankylosing Spondylitis; ASAS: Assessment of SpondyloArthritis international Society; BASRI: Bath Ankylosing Spondylitis Radiology Index; mSASSS: Modified Stoke Ankylosing Spondylitis Spine Score; OASIS: Outcome in AS International Study; OMERACT: Outcome Measures in Rheumatology Clinical Trials; RASSS: Radiographic Ankylosing Spondylitis Spinal Score; SASSS: Stoke AS Spine Score; SD: standard deviation; SDC: smallest detectable change; SpA: spondyloarthritis; VC: vertebral corner.

## Competing interests

The authors declare that they have no competing interests.

## Authors' contributions

SR, AvT, RL and DvdH designed the study. SR, AvT, CS, RL, FvdB, MD and DvdH collected the data, SR and CS read the radiographs. SR, AvT, RL and DvdH analysed the data and critically interpreted the results. SR prepared the first version of the manuscript. All the authors reviewed the draft versions and gave their approval of the final version of the manuscript.

## References

[B1] van der Heijde D Calin A Dougados M Khan MA van der Linden S Bellamy N Selection of instruments in the core set for DC-ART, SMARD, physical therapy, and clinical record keeping in ankylosing spondylitis. Progress report of the ASAS Working Group. Assessments in Ankylosing Spondylitis J Rheumatol 1999 26 951 954 10229426

[B2] Machado P Landewe R Braun J Hermann KG Baker D van der Heijde D Both structural damage and inflammation of the spine contribute to impairment of spinal mobility in patients with ankylosing spondylitis Ann Rheum Dis 2010 69 1465 1470 10.1136/ard.2009.124206 20498215

[B3] Wanders A Landewe R Dougados M Mielants H van der Linden S van der Heijde D Association between radiographic damage of the spine and spinal mobility for individual patients with ankylosing spondylitis: can assessment of spinal mobility be a proxy for radiographic evaluation? Ann Rheum Dis 2005 64 988 994 10.1136/ard.2004.029728 15958757 PMC1755579

[B4] Landewe R Dougados M Mielants H van der Tempel H van der Heijde D Physical function in ankylosing spondylitis is independently determined by both disease activity and radiographic damage of the spine Ann Rheum Dis 2009 68 863 867 10.1136/ard.2008.091793 18628283

[B5] Zochling J van der Heijde D Burgos-Vargas R Collantes E Davis JC Jr Dijkmans B Dougados M Geher P Inman RD Khan MA Kvien TK Leirisalo-Repo M Olivieri I Pavelka K Sieper J Stucki G Sturrock RD van der Linden S Wendling D Bohm H van Royen BJ Braun J ASAS/EULAR recommendations for the management of ankylosing spondylitis Ann Rheum Dis 2006 65 442 452 10.1136/ard.2005.041137 16126791 PMC1798102

[B6] Braun J van den Berg R Baraliakos X Boehm H Burgos-Vargas R Collantes-Estevez E Dagfinrud H Dijkmans B Dougados M Emery P Geher P Hammoudeh M Inman RD Jongkees M Khan MA Kiltz U Kvien T Leirisalo-Repo M Maksymowych WP Olivieri I Pavelka K Sieper J Stanislawska-Biernat E Wendling D Ozgocmen S van Drogen C van Royen B van der Heijde D 2010 update of the ASAS/EULAR recommendations for the management of ankylosing spondylitis Ann Rheum Dis 2011 70 896 904 10.1136/ard.2011.151027 21540199 PMC3086052

[B7] MacKay K Mack C Brophy S Calin A The Bath Ankylosing Spondylitis Radiology Index (BASRI): a new, validated approach to disease assessment Arthritis Rheum 1998 41 2263 2270 10.1002/1529-0131(199812)41:12<2263::AID-ART23>3.0.CO;2-I 9870884

[B8] Averns HL Oxtoby J Taylor HG Jones PW Dziedzic K Dawes PT Radiological outcome in ankylosing spondylitis: use of the Stoke Ankylosing Spondylitis Spine Score (SASSS) Br J Rheumatol 1996 35 373 376 10.1093/rheumatology/35.4.373 8624642

[B9] Creemers MC Franssen MJ van't Hof MA Gribnau FW van de Putte LB van Riel PL Assessment of outcome in ankylosing spondylitis: an extended radiographic scoring system Ann Rheum Dis 2005 64 127 129 10.1136/ard.2004.020503 15051621 PMC1755183

[B10] Wanders AJ Landewe RB Spoorenberg A Dougados M van der Linden S Mielants H van der Tempel H van der Heijde DM What is the most appropriate radiologic scoring method for ankylosing spondylitis? A comparison of the available methods based on the Outcome Measures in Rheumatology Clinical Trials filter Arthritis Rheum 2004 50 2622 2632 10.1002/art.20446 15334477

[B11] van der Heijde D Landewe R Selection of a method for scoring radiographs for ankylosing spondylitis clinical trials, by the Assessment in Ankylosing Spondylitis Working Group and OMERACT J Rheumatol 2005 32 2048 2049 16206368

[B12] Baraliakos X Listing J Rudwaleit M Sieper J Braun J Development of a radiographic scoring tool for ankylosing spondylitis only based on bone formation: addition of the thoracic spine improves sensitivity to change Arthritis Rheum 2009 61 764 771 10.1002/art.24425 19479705

[B13] Boers M Brooks P Strand CV Tugwell P The OMERACT filter for Outcome Measures in Rheumatology J Rheumatol 1998 25 198 199 9489805

[B14] Spoorenberg A van der Heijde D de Klerk E Dougados M de Vlam K Mielants H van der Tempel H van der Linden S Relative value of erythrocyte sedimentation rate and C-reactive protein in assessment of disease activity in ankylosing spondylitis J Rheumatol 1999 26 980 984 10229432

[B15] Spoorenberg A de Vlam K van der Heijde D de Klerk E Dougados M Mielants H van der Tempel H Boers M van der Linden S Radiological scoring methods in ankylosing spondylitis: reliability and sensitivity to change over one year J Rheumatol 1999 26 997 1002 10229436

[B16] Bland JM Altman DG Statistical methods for assessing agreement between two methods of clinical measurement Lancet 1986 1 307 310 2868172

[B17] Bruynesteyn K Boers M Kostense P van der Linden S van der Heijde D Deciding on progression of joint damage in paired films of individual patients: smallest detectable difference or change Ann Rheum Dis 2005 64 179 182 10.1136/ard.2003.018457 15286006 PMC1755378

[B18] van Tubergen A Ramiro S van der Heijde D Dougados M Mielants H Landewe R Development of new syndesmophytes and bridges in ankylosing spondylitis and their predictors: a longitudinal study Ann Rheum Dis 2012 71 518 523 10.1136/annrheumdis-2011-200411 21989544

[B19] Poddubnyy D Haibel H Listing J Marker-Hermann E Zeidler H Braun J Sieper J Rudwaleit M Baseline radiographic damage, elevated acute-phase reactant levels, and cigarette smoking status predict spinal radiographic progression in early axial spondylarthritis Arthritis Rheum 2012 64 1388 1398 10.1002/art.33465 22127957

[B20] Baraliakos X Listing J von der Recke A Braun J The natural course of radiographic progression in ankylosing spondylitis--evidence for major individual variations in a large proportion of patients J Rheumatol 2009 36 997 1002 10.3899/jrheum.080871 19332632

[B21] van der Heijde D Landewe R Baraliakos X Houben H van Tubergen A Williamson P Xu W Baker D Goldstein N Braun J Radiographic findings following two years of infliximab therapy in patients with ankylosing spondylitis Arthritis Rheum 2008 58 3063 3070 10.1002/art.23901 18821688

[B22] van der Heijde D Landewe R Einstein S Ory P Vosse D Ni L Lin SL Tsuji W Davis JC Jr Radiographic progression of ankylosing spondylitis after up to two years of treatment with etanercept Arthritis Rheum 2008 58 1324 1331 10.1002/art.23471 18438853

[B23] van der Heijde D Salonen D Weissman BN Landewe R Maksymowych WP Kupper H Ballal S Gibson E Wong R Assessment of radiographic progression in the spines of patients with ankylosing spondylitis treated with adalimumab for up to 2 years Arthritis Res Ther 2009 11 R127 10.1186/ar2794 19703304 PMC2745811

[B24] Wanders A Landewe R Spoorenberg A de Vlam K Mielants H Dougados M van der Linden S van der Heijde D Scoring of radiographic progression in randomised clinical trials in ankylosing spondylitis: a preference for paired reading order Ann Rheum Dis 2004 63 1601 1604 10.1136/ard.2004.022038 15297280 PMC1754835

